# Odontography; or a Treatise on the Comparative Anatomy of the Teeth; Their Physiological Relations, Mode of Development, and Microscopic Structure, in the Vertebrate Animals

**Published:** 1840-07

**Authors:** 


### Art. IX.

*Odontography; or a Treatise on the Comparative Anatomy of the Teeth; their Physiological Relations, Mode of Development, and Microscopic Structure, in the Vertebrate Animals. Illustrated by upwards of* 150 *Plates.* By Richard Owen, F.R.S. Correspondent of the Royal Academy of Sciences of Paris, Berlin, &c. &c., Hunterian Professor to the Royal College of Surgeons, London. Part I.—*London,* 1840. 8vo, pp. 112. Pl. 50.

The "decline of science in England" was, a few years ago, the subject of much discussion, both at home and abroad. To what extent the imputation was, at the time, well-founded we shall not stop to enquire, being satisfied that it can no longer be cast on our country either by native malcontents or foreign detractors. Had we nothing else to show, the splendid work of which the first part is now before us would be sufficient. It embodies the results of a discovery which alone stamps its author as the most original contributor to anatomical science at the present day; whilst, in the manner in which it has been worked out, there is abundant proof of his being the most philosophic.

It is not our present purpose to enter upon the details of this interesting subject; it will be better, on every account, that we should reserve them until the completion of the work. But we think this a fit opportunity to direct the attention of our readers to the nature and value of the researches whose fruits will be embodied in it. And we do this the more willingly because the author has reserved his general introduction until the whole of the details have been exposed; with the very proper motive, we presume, of adapting it most advantageously to the new facts with which he may become acquainted during the course of publication. With his characteristic modesty, he confines his statements to mere facts; and, without any pompous heraldry, presents them in their simplest dress, and, to some perhaps, their least attractive form. But to those who are prepared to appreciate their value, the idea of "beauty when unadorned adorned the most," will doubtless occur, as it has to us; for never has our *beau ideal* of the manner in which an important discovery should be communicated to the world been so completely realized.

The most important *general facts* discovered and substantiated by Professor Owen regarding the structure of the teeth, we believe to be the two following:

1. That teeth grow, like bone, by intussusception; that they are not extra-vascular structures, as Hunter, followed by Cuvier and many others, maintained; but that they are originally formed by the deposition of calcareous matter in cellular tissue, by a process bearing a general analogy to that of ossification.

2. That the microscopic examination of the teeth reveals correspondences and differences in their structure in the various groups of vertebrated animals, so constant and easily recognized that from the smallest fragment of a fossil as well as recent tooth, not only the class and order but even the family, and in some instances the nearest allied genus of the animal to which the tooth belonged, may be predicated with certainty.

The first of these discoveries was made by observation of the development of the teeth in the fœtal shark, in 1838. Mr. Owen remarks that in these, as in many other fishes, we have an exemplification on a large scale of the earliest or papillary stage of dental development in the higher classes of animals. It is not succeeded by either a follicular or an eruptive stage; since the formative papillæ are never inclosed, and consequently never break forth. The unossified pulps, examined with a high power, consist of semi-opaque granules or cells, suspended in a clear *matrix;* and the whole is inclosed in a tough transparent membrane which forms the outer surface of the pulp. The formation of the tooth commences by the deposition of earthy particles in the latter, which thus becomes the enamel-like polished coating of the tooth; and the process of calcification gradually extends from without inwards, the pulpy substance being actually converted into solid *dentine;* and not giving place to excreted layers of it, as commonly supposed.

It is among the highest characteristics of the true philosopher to determine where he may safely and certainly reason from analogy, and to distinguish the cases in which he must distrust it. The celebrated assertion of Newton, that the diamond was combustible, is familiar to every one; though few have perceived as he did the importance of the analogy on which he rested it. Now on the single series of observations to which we have referred, Mr. Owen has based his general statement of the character of the process of *dentification* in all vertebrated animals; and the event has proved him to be right. The independent observations of Schwann upon the development of mammalian teeth, which we have already noticed (vol. ix. p. 513) lead to precisely the same conclusion, when interpreted by those of Mr. Owen. But without the latter, their full bearing might not have been recognized; and this has indeed happened even to Müller, who, in the second edition of his *Physiologie*, (in which he takes full cognizance of his friend Schwann's excellent observations,) still classes the formation of teeth with that of hair and other extra-vascular parts, in the category of "Formation by Apposition." Mr. Owen's views were expounded in his lectures at the College of Surgeons in May, 1839, previously to his becoming acquainted with Schwann's researches; and a more detailed statement of them was transmitted by him to the French Academy, in acknowledgment of their election of him as correspondent; which statement will be found in the *Comptes Rendus* for last year.

If this principle is sound, as we think there can be no doubt that it is, and comes, as it must, to be generally received, it is the most important information on the subject of the teeth that can be given to the practical dentist. To prove that teeth are essentially developed like bone,—to show by what modifications of osseous structure their vitality, when fully formed, is so masked that they have appeared to the most intelligent anatomists as brute bodies, and to point out by what channels a tooth, though in the main extra-vascular, may receive materials for its support, or influences to its decay—are among the objects of Mr. Owen's work; and it is obvious that the settlement of these questions must have a most important influence on the progress of dental pathology.

To the medical man *this* is the point of most importance; but to the scientific anatomist, and more especially to the palæontological naturalist, the *second* is of surpassing interest. The fame of Cuvier will probably in future years chiefly rest upon the discovery that there is such a degree of conformity between the various parts and organs of an animal, that the whole may be inferred, by an anatomist rendered skilful by previous knowledge, from a small portion. But it was necessary that this portion should retain its external form, and should belong to some characteristic part of the structure. That conclusions, equal in certainty and minuteness, should be drawn from an amorphous fragment was never dreamed of by the illustrious author of the Ossemens Fossiles; and we cannot help regarding this discovery as yet more remarkable than any *single* principle established by Cuvier. The application of it has served to set at rest prolonged discussions founded upon the external appearance of most characteristic portions of the skeleton; and thus the value of this means of investigation is shown to be superior to that of any other. A few instances must suffice.

Most of our readers, we presume, are acquainted with the existence of a certain fossil in the Stonesfield slate which has been commonly regarded, after Cuvier, as having belonged to a marsupial quadruped, but which many eminent osteologists pronounced to be the remains of a reptile. Not many months ago a very vigorous war was carried on between the two parties, and many new names were proposed for the creature, in accordance with the respective views of the nomenclators. Amongst others the name *Botheratio-therium* was jocularly conferred upon it by the reporter of the Proceedings of the Geological Society in the Athenæum, and gravely adopted by the French savans. Mr. Owen was the chief supporter of Cuvier's views; and against him were ranged Blainville, Grant, Ogilby, and other eminent comparative anatomists. The latter based some of their most important arguments on the supposed analogy of the *Basilisaurus*, recently discovered by Dr. Harlan of Philadelphia, which had, like the Stonesfield fossil, double-fanged teeth,—a character previously supposed peculiar to mammalia. But being led by other appearances to doubt the saurian nature of these remains, Mr. Owen submitted a section of a tooth to microscopic examination; and the result confirmed his previous views, by demonstrating its place to be between the carnivorous and herbivorous cetacea, as Dr. Harlan then readily admitted. Thus the analogy which had been so much insisted on proved to be as much in Mr. Owen's favour as it before seemed against him; and the marsupial character of the Stonesfield fossil is now, we believe, generally admitted.

A similar confirmation has been afforded by the microscopic examination of the teeth to Mr. Owen's opinion, formed upon independent grounds, in opposition to those of Blainville and other eminent anatomists, that Cuvier was right in regarding the *megatherium* as more closely allied to the sloths and ant-eaters than to the armadillo; and that the tesselated armour, found in occasional proximity with its remains, does not belong to it, but to a distinct genus, to which the name *Glyptodon* has been given by Mr. Owen, more resembling the armadillo.

It would be easy to multiply instances of this kind; but we forbear for the present to do so. We must remark, however, that the entire working-out of the principle above alluded to, as well as the first conception of it, is due to Mr. Owen; and this has been accomplished by the laborious and minute examination and comparison of several hundred microscopic sections of recent and fossil teeth, the most important of which will be figured in this work. The plates of the part already published leave nothing to be desired in regard to illustration; and we trust that the demand for the work will be such as to convince its author that his merits, as the most eminent comparative anatomist of his day—the worthy successor of Hunter and Cuvier, combining the original and philosophic spirit of the one with the industrious mastery of details which was the peculiar characteristic of the other—are not lost sight of by his countrymen. The members of the College of Surgeons may feel an honest pride in having contributed to develope his genius, and in retaining in their noble museum its laborious results. We trust that this treatise is only the forerunner of similar works on other departments of comparative anatomy; and that the osseous, genital, and other important systems may be treated with the same skill and originality, and in the same self-contained form, so that if health and time be not spared him to complete the whole, each portion may be complete in itself.

